# Analysis of the correlation between physical activity level, sleep quality, and anxiety levels in middle-aged and older adults: a cross-sectional study

**DOI:** 10.3389/fpubh.2025.1629695

**Published:** 2025-10-30

**Authors:** Bin Tang, Ying Hu, Chuanjie Xu, Nana Wang, Yu Li, Jianping Li

**Affiliations:** Department of Neurological Intensive Care Unit, The First People’s Hospital of Zunyi (Third Affiliated Hospital of Zunyi Medical University), Zunyi, China

**Keywords:** physical activity, sleep quality, anxiety, association, cross sectional study

## Abstract

**Objective:**

This study aimed to investigate the associations between physical activity levels, sleep quality, and anxiety status in a cross-sectional sample of middle-aged and older adults.

**Materials and methods:**

Anxiety was assessed using the Generalized Anxiety Disorder 7 (GAD-7) scale, and sleep quality was evaluated using the Pittsburgh Sleep Quality Index (PSQI). Physical activity levels were evaluated using the International Physical Activity Questionnaire (IPAQ). Multivariable logistic regression models were used to analyze the associations, adjusting for potential confounders such as age, gender, body mass index (BMI), smoking, and alcohol use.

**Results:**

A total of 488 participants were included. Both good sleep quality and adherence to recommended physical activity levels were independently associated with a significantly reduced risk of anxiety (*p* < 0.001). Sleep quality, rather than sleep duration, emerged as a key factor of anxiety. Stratified analyses showed that these associations were more pronounced in females, non-smokers, and urban residents.

**Conclusion:**

This study underscores the importance of sleep quality and physical activity in reducing anxiety risk. These findings suggest that integrated strategies promoting better sleep and increased physical activity may be effective for anxiety prevention and intervention among middle-aged and older adults.

## 1 Introduction

Anxiety is a common mental health disorder characterized by persistent and excessive worry, often accompanied by autonomic symptoms such as palpitations, rapid breathing, and sweating (1). Its high global prevalence and substantial impact on daily functioning, productivity, and social relationships have drawn increasing attention in recent years (2). Empirical evidence suggests that rapid socioeconomic changes, increased job demands, and longer working hours are associated with higher anxiety prevalence, reflecting the influence of modern societal pressures on mental health (3). These trends underscore the need for effective prevention and intervention strategies. Middle-aged and older adults are particularly relevant to this discussion, as aging-related physiological changes and the higher prevalence of chronic illnesses in these populations may heighten vulnerability to both anxiety and sleep disturbances (4).

Sleep quality is closely linked to anxiety risk, and interventions improving sleep often yield mental health benefits. Both short (<7 h) and long (≥9 h) sleep durations have been associated with elevated anxiety risk (5), while chronic sleep deprivation may impair emotional regulation and exacerbate symptoms (6). Age appears to moderate this relationship: younger adults may be more sensitive to sleep loss than older adults (7).

Physical activity supports cardiovascular, metabolic, and mental health (8). WHO recommends at least 600 MET-min/week of moderate-to-vigorous activity, associated with about 30% lower anxiety risk (9). Mechanisms may include reduced inflammation and regulation of mood-related neurotransmitters (10). However, effects vary by type and intensity; high-intensity exercise may increase anxiety in some cases (11), and individual habits and baseline symptoms may influence outcomes.

Despite numerous studies examining the relationships between sleep, physical activity, and anxiety, most research has focused on these factors individually. This study aimed to explore the independent effects of sleep quality and physical activity on anxiety, aligning with the scope of the analysis. For example, exercise can improve sleep quality, which in turn may indirectly alleviate anxiety symptoms (12). However, this mechanism has not been thoroughly investigated. Furthermore, factors such as age, gender, BMI, and lifestyle habits (e.g., smoking, alcohol consumption) may moderate these relationships, adding complexity to the research (13). Therefore, a systematic analytical approach is necessary to comprehensively assess the interactions among physical activity, sleep quality, and anxiety, while exploring potential moderating factors.

Based on these considerations, the present study adopted a cross-sectional design to investigate the relationships among physical activity levels, sleep quality, and anxiety symptoms. Sleep quality, anxiety, and physical activity were assessed using the Pittsburgh Sleep Quality Index (PSQI), the Generalized Anxiety Disorder-7 (GAD-7) scale, and the International Physical Activity Questionnaire (IPAQ), respectively. Demographic and lifestyle variables were also collected to provide a broader context for these associations. The null hypothesis proposed that poor sleep quality and low physical activity levels are associated with more severe anxiety symptoms. The findings aim to contribute empirical evidence to support anxiety prevention and intervention efforts and inform the development of targeted health promotion strategies.

## 2 Methods

### 2.1 Research sample

This study was approved by the Ethics Committee of Zunyi Medical University (Ethical Approval No.: 2025-1-111) and strictly adhered to the ethical principles outlined in the Declaration of Helsinki (14). The study employed a cross-sectional design, and all participants were fully informed about the study’s objectives, procedures, and potential risks before the study commenced, and provided written informed consent.

Inclusion criteria: (1) Adults aged 45 years and older; capable of understanding and agreeing to participate in the study and signing the informed consent form. (2) Residing in a relatively stable living environment for at least 1 month (to avoid the impact of short-term environmental changes on sleep and physical activity). (3) Capable of independently completing assessment tools such as the PSQI, GAD-7, and IPAQ; no severe physical illnesses or motor function disabilities, and able to perform daily physical activities. (4) No systemic psychological treatment or use of psychotropic medications affecting anxiety or sleep in the past 3 months. Exclusion criteria: (1) Diagnosed with severe mental disorders (e.g., schizophrenia, bipolar disorder). (2) Severe sleep disorders such as insomnia or sleep apnea syndrome (diagnosed). (3) Serious chronic diseases affecting physical activity (e.g., cardiovascular diseases, severe joint diseases). (4) Recent major life events (e.g., bereavement, significant financial crisis) that may impact anxiety levels, pregnant or breastfeeding women. (5) Recent (within the past 3 months) history of drug or alcohol abuse. (6) Unwilling or unable to cooperate with completing relevant questionnaires during the study.

### 2.2 Sleep assessment: PSQI

The Pittsburgh PSQI was used to assess the sleep quality of participants in this study. The PSQI, developed by Buysse et al. (15), consists of 19 self-reported items and covers seven dimensions: subjective sleep quality, sleep latency, sleep duration, habitual sleep efficiency, sleep disturbances, use of sleep medication, and daytime dysfunction (16). The score for each dimension ranges from 0 to 3, and the total score ranges from 0 to 21, with higher scores indicating poor sleep quality.

In addition, to further analyze sleep patterns, participants were asked to respond to a self-assessment question about sleep duration: “How many hours do you typically sleep on workdays or weeknights?” Based on national sleep duration recommendations, participants were categorized into three groups based on their sleep duration: short sleep (<7 h), normal sleep (7–9 h), and long sleep (≥9 h) (17). This classification method helps to further explore the relationship between different sleep durations, anxiety levels, and physical activity levels.

### 2.3 Physical activity assessment

The short form of the IPAQ was used to assess participants’ physical activity levels. Participants were asked to recall and report the frequency and duration of high-intensity physical activity (HPA), moderate-intensity physical activity (MPA), walking (W), and sedentary behavior during the past 7 days. The physical activity levels were calculated in terms of Metabolic Equivalent of Task (MET), which estimates the energy expenditure for various activities. The specific MET values are as follows: walking = 3.3, moderate-intensity physical activity = 4.0, and high-intensity physical activity = 8.0. The total physical activity index was calculated by summing the METs expended on walking, moderate-intensity physical activity, and high-intensity physical activity (18). According to the WHO physical activity recommendations, individuals whose total MET-minutes per week (MET-min/week) are considered to meet the physical activity standards. Based on the IPAQ classification, participants were divided into two categories: “meeting the standard” (≥600 MET-min/week) and “not meeting the standard” (<600 MET-min/week).

### 2.4 Anxiety assessment: GAD-7

Anxiety levels were assessed using the Generalized Anxiety Disorder 7 (GAD-7) scale. The scale consists of 7 items, each rated on a 4-point Likert scale (0–3 points), with a total score range from 0 to 21 (19). Based on the total score, anxiety levels were categorized as follows: no anxiety (0–4 points), mild anxiety (5–9 points), moderate anxiety (10–14 points), and severe anxiety (15–21 points). The GAD-7 has demonstrated good reliability and validity, and its Chinese version has been validated in Chinese populations (20).

### 2.5 Control of confounding factors

Demographic information of the participants, including age, gender, body mass index (BMI), smoking status (yes/no), alcohol consumption status (yes/no), history of hypertension (yes/no), and history of diabetes (yes/no), was collected via questionnaires. BMI was calculated using the standard formula: BMI = weight (kg) / height^2^ (m^2^). During the data analysis, these variables were included in the statistical models to control for potential confounding factors, thus reducing the impact of these factors on the relationship between anxiety levels, sleep quality, and physical activity index, thereby enhancing the reliability of the study’s conclusions.

### 2.6 Statistical analysis

All data were initially recorded by the research team members using paper questionnaires, which were then transcribed into an electronic database. Questionnaire data were exported from the online survey platform, and scores for each indicator were calculated, followed by data organization and cleaning. Data entry and management were performed using Microsoft Excel, and statistical analyses were conducted using SPSS 27.0 (IBM) and GraphPad Prism 8.0.2 (GraphPad Software).

Descriptive statistics were used to summarize the basic characteristics of the study population, with categorical variables presented as frequencies and percentages, and continuous variables expressed as mean ± standard deviation (Mean ± SD) or median (interquartile range, IQR). Before conducting parametric tests, the distribution of continuous variables was assessed for normality using the Shapiro–Wilk test, and homogeneity of variances was examined using Levene’s test. Chi-square tests were used to compare differences between categorical variables, exploring the relationships between different levels of physical activity, sleep quality, and anxiety status. For comparisons of continuous variables between groups, independent t-tests (for normally distributed data) or Mann–Whitney *U* tests (for non-normally distributed data) were used. Multivariable logistic regression models were employed to assess the associations between physical activity levels, sleep quality, and anxiety status, adjusting for potential confounders. In the regression models, anxiety level was the dependent variable, and physical activity level and sleep quality were the main independent variables, while age, gender, BMI, smoking, and alcohol consumption were included as covariates. The results of the models were presented as odds ratios (OR) with 95% confidence intervals (95% CI), with *p* < 0.05 considered statistically significant.

## 3 Results

### 3.1 Baseline information for participants

**Figure 1:**
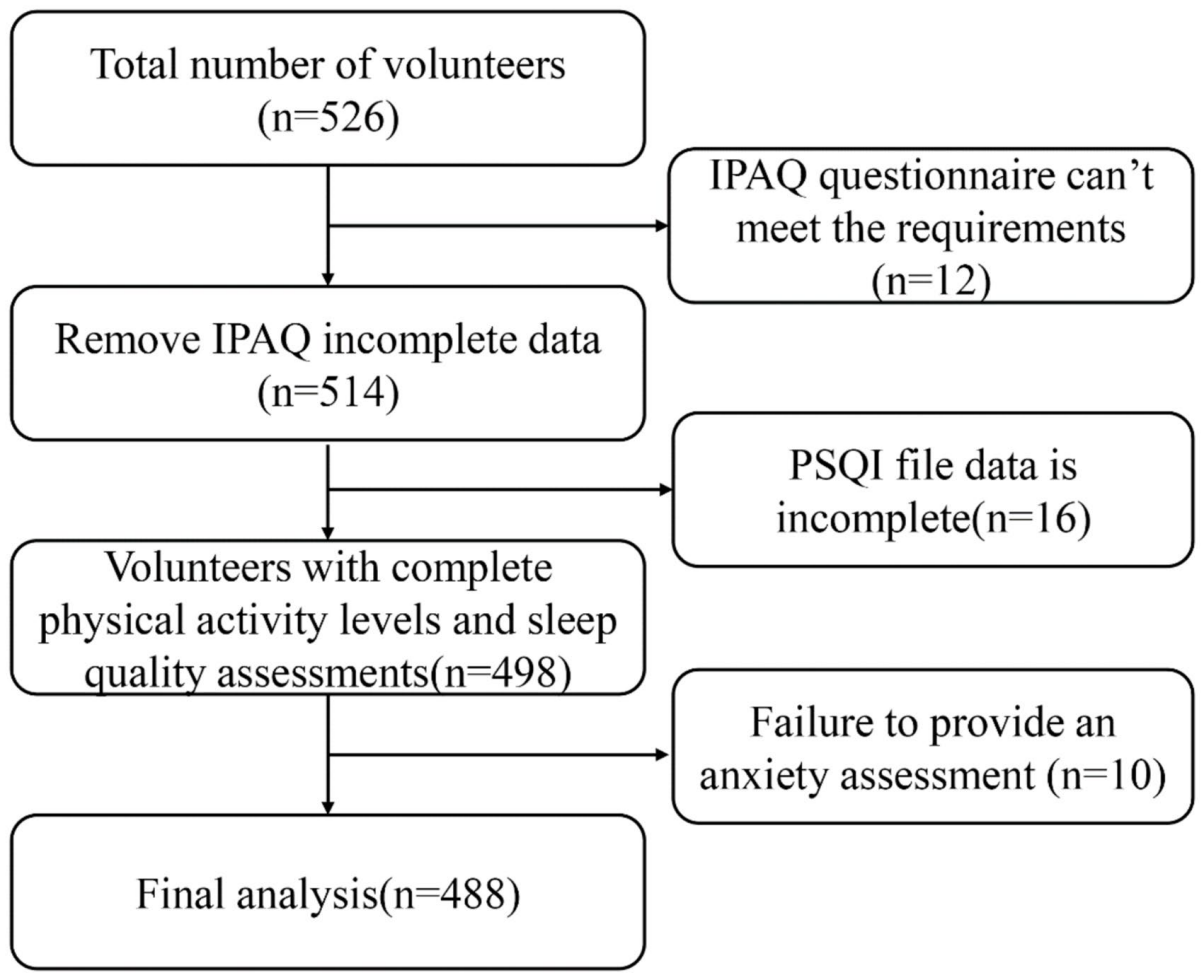
Flowchart of participant recruitment, exclusion criteria, and final sample included in the analysis.

A total of 526 volunteers were recruited for this study. After screening, 12 participants with invalid IPAQ questionnaires, 16 participants with incomplete PSQI data, and 10 participants who did not provide anxiety assessments were excluded, resulting in 488 participants included in the final analysis (Figure 1). Based on the GAD-7 scoring criteria, 122 (25.0%) were classified into the anxiety group and 366 (75.0%) into the non-anxiety group.

Table 1 presents the demographic characteristics of the study sample. In the overall sample (*n* = 488), there were slightly more females than males (52.0% vs. 48.0%), with an average age of 57.82 ± 10.22 years. Most participants resided in urban areas (74.0%), were married (85.0%), and had an education level below high school (83.6%). Regarding lifestyle habits, 65.6% of participants were non-smokers, and 68.0% did not drink alcohol. Regarding health status, 44.5% of participants reported a history of hypertension, 10.9% reported a history of diabetes, and 24.2% were overweight or obese (BMI ≥ 24 kg/m^2^).

**Table 1:** Sociodemographic characteristics of participants by anxiety status (*n* = 488).

Characteristic	All (*n* = 488) (weighted column %)	Non-anxiety *N* (%)	Anxiety *N* (%)	*P*
Gender				0.917
Male	234(48.0)	175(47.8)	59(48.4)	
Female	254(52.0)	191(52.2)	63(51.6)	
Age	57.82 ± 10.22	57.44 ± 9.70	58.96 ± 11.63	0.217
Region of residence				0.858
Countryside	127(26.0)	96(26.2)	31(25.4)	
Cities	361(74.0)	270(73.8)	91(74.6)	
Marital status				0.164
Married	415(85.0)	316(86.3)	99(81.1)	
Other	73(15.0)	50(13.7)	23(18.9)	
Education				0.778
Below secondary school	408(83.6)	307(83.9)	101(82.8)	
Secondary school and above	80(16.4)	59(16.1)	21(17.2)	
Smoking				0.379
No	320(65.6)	236(64.5)	84(68.9)	
Yes	168(34.4)	130(35.5)	38(31.1)	
Drinking				0.262
No	332(68.0)	244(66.7)	88(72.1)	
Yes	156(32.0)	122(33.3)	34(27.9)	
High blood pressure				0.494
No	271(55.5)	200(54.6)	71(58.2)	
Yes	217(44.5)	166(45.4)	51(41.8)	
Diabetes				0.208
No	435(89.1)	330(90.2)	105(86.1)	
Yes	53(10.9)	36(9.8)	17(13.9)	
BMI level				0.272
Light or normal	370(75.8)	282(77.0)	88(72.1)	
Overweight and obese	118(24.2)	84(23.0)	34(27.9)	

Continuous variables are expressed as mean ± SD; categorical variables as *n* (%).

When comparing the demographic characteristics between the anxiety group and the non-anxiety group, no statistically significant differences were found between the two groups in terms of gender (*p* = 0.917), age (*p* = 0.217), residential area (*p* = 0.858), marital status (*p* = 0.164), education level (*p* = 0.778), smoking status (*p* = 0.379), drinking status (*p* = 0.262), history of hypertension (*p* = 0.494), history of diabetes (*p* = 0.208), and BMI (*p* = 0.272). These results suggest that the demographic distribution of the anxiety and non-anxiety groups in this study was well-balanced, providing a good foundation for subsequent analyses.

### 3.2 Independent associations between physical activity, sleep, and anxiety

**Figure 2:**
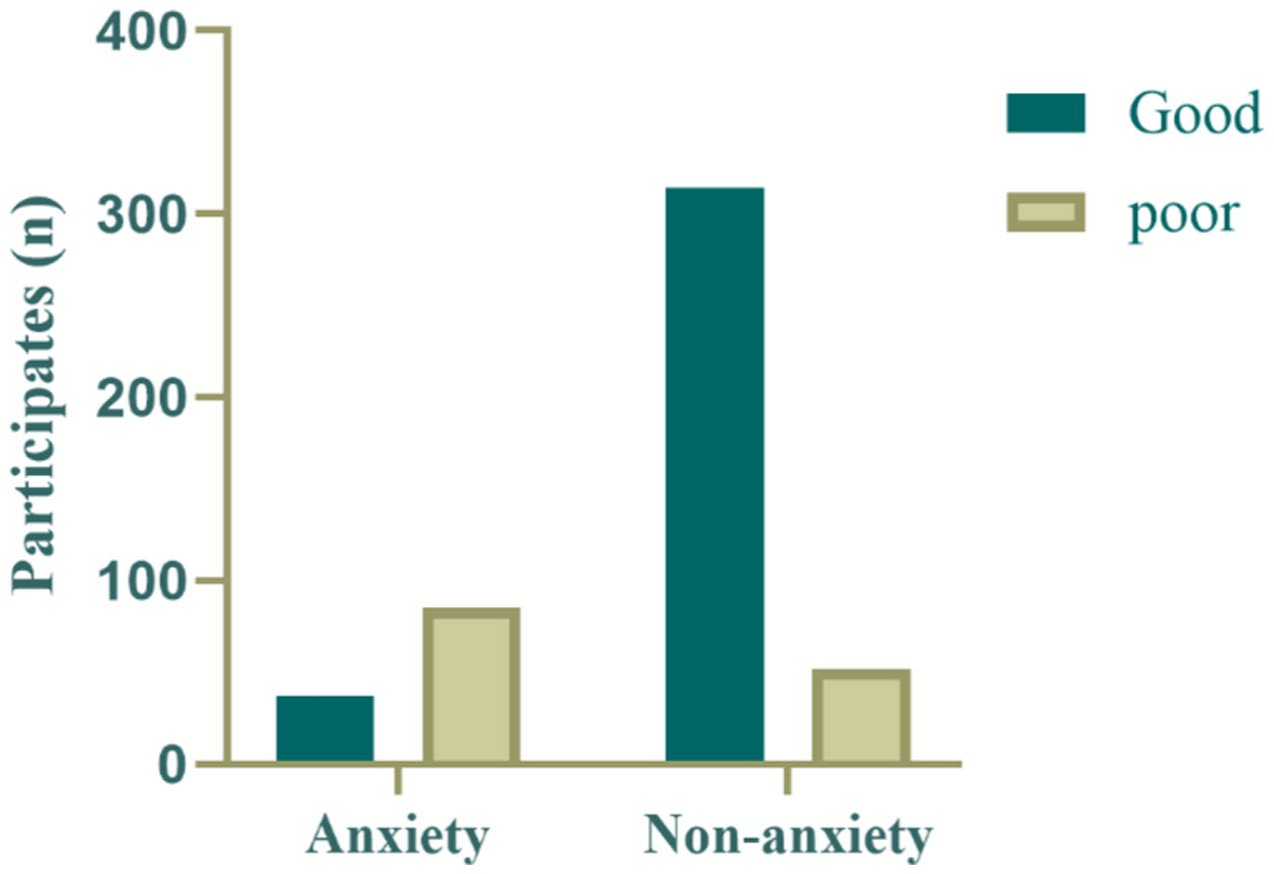
Distribution of sleep quality levels in the anxiety group and non-anxiety group.

**Figure 3:**
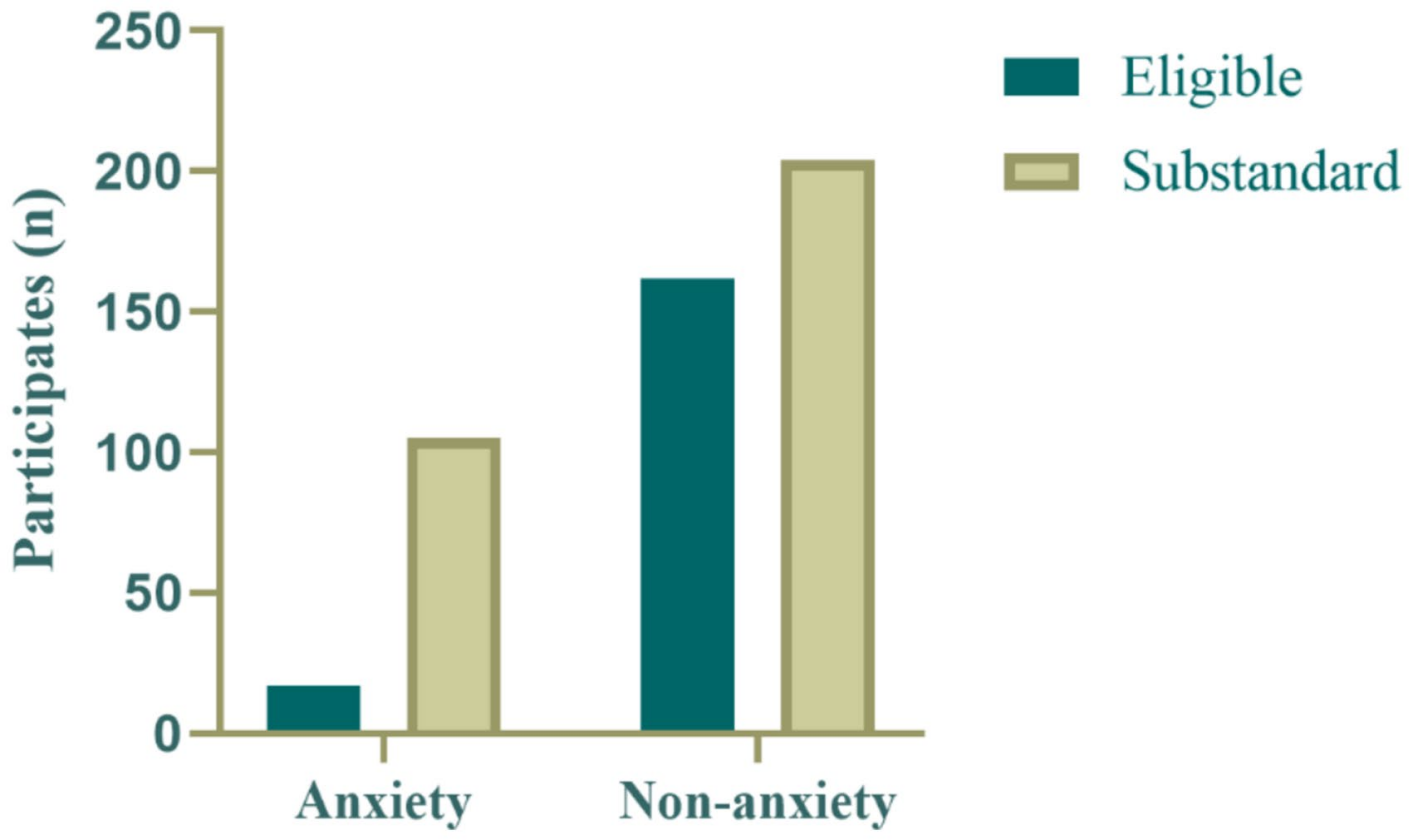
Distribution of physical activity levels in the anxiety group and non-anxiety group.

Figures 2, 3 show the distributions of sleep quality and physical activity levels between the anxiety and non-anxiety groups. In the anxiety group, the majority of participants (*n* = 85) exhibited poor sleep quality, while only a few (*n* = 37) had good sleep quality. In contrast, in the non-anxiety group, most participants (*n* = 314) had good sleep quality, with a smaller proportion (*n* = 52) experiencing poor sleep quality. This suggests a close association between anxiety status and sleep quality, with individuals in the anxiety group being more likely to experience sleep problems (Figure 2).

Regarding physical activity levels, only a few participants in the anxiety group (*n* = 17) met the WHO recommended physical activity standards (≥600 MET-min/week), while most participants (*n* = 105) did not meet the standard. In the non-anxiety group, although the majority of participants (*n* = 204) did not meet the recommended physical activity levels, the proportion of those meeting the standard (*n* = 162) was significantly higher than in the anxiety group. This finding suggests that physical activity levels are also associated with anxiety status, supporting the hypothesis that physical activity may have a protective effect against anxiety (Figure 3).

Univariate logistic regression analysis confirmed significant associations between physical activity levels and anxiety status (*p* < 0.001) (Table 2). Among all participants (*n* = 488), 63.3% (309 participants) did not meet the recommended physical activity levels (<600 MET-min/week), while 36.7% (179 participants) met the standard. The proportion of participants in the anxiety group who did not meet the physical activity standard (86.1%) was significantly higher than in the non-anxiety group (55.7%).

**Table 2:** A univariate logistic regression analysis of physical activity levels and sleep quality on anxiety.

Characteristic	All (*n* = 488) (weighted column %)	Non-anxiety *N* (%)	Anxiety *N* (%)	*P*
IPAQ				<0.001
Substandard	309(63.3)	204(55.7)	105(86.1)	
Eligible	179(36.7)	162(44.3)	17(13.9)	
Quality of sleep				<0.001
Good	351(71.9)	314(85.8)	37(30.3)	
Poor	137(28.1)	52(14.2)	85(69.7)	
Sleep duration	6.51 ± 2.18	6.45 ± 2.16	6.72 ± 2.21	0.172

For sleep quality, 71.9% (351 participants) had good sleep quality, while 28.1% (137 participants) had poor sleep quality. The proportion of poor sleep quality was markedly higher in the anxiety group (69.7%) than in the non-anxiety group (14.2%) (*p* < 0.001). Sleep duration did not differ significantly between groups: 6.72 ± 2.21 h in the anxiety group and 6.45 ± 2.16 h in the non-anxiety group (*p* = 0.172). Poor sleep quality and insufficient physical activity were each strongly associated with higher anxiety risk, whereas sleep duration showed no significant relationship.

### 3.3 Associations between physical activity, sleep, and anxiety

To more comprehensively assess the associations between physical activity levels, sleep quality, and anxiety status while controlling for potential confounders, multivariable logistic regression analysis was performed. The analysis included both unadjusted and adjusted models (adjusting for confounders such as age, gender, BMI, smoking, alcohol consumption, hypertension, and diabetes) (Table 3).

**Table 3:** Multifactorial logistic regression analysis of physical activity levels and sleep quality on anxiety.

Characteristic	Unadjusted Model OR (95% CI)	*P*	Adjusted Model OR (95% CI)	*P*
Quality of sleep		<0.001		<0.001
Poor	1 (Ref)		1 (Ref)	
Good	0.069(0.041–0.115)		0.065(0.038–0.111)	
IPAQ		<0.001		<0.001
Eligible	1 (Ref)		1 (Ref)	
Substandard	5.345(2.849–10.027)		5.408(2.827–10.344)	

In the unadjusted model, compared with poor sleep quality, good sleep quality was associated with a markedly reduced risk of anxiety (OR = 0.069, 95% CI: 0.041–0.115, *p* < 0.001). After adjusting for potential confounders, the association remained significant and similar in magnitude (OR = 0.065, 95% CI: 0.038–0.111, *p* < 0.001).

For physical activity, in the unadjusted model, not meeting the WHO recommended standard was associated with substantially higher anxiety risk compared with meeting the standard (OR = 5.345, 95% CI: 2.849–10.027, *p* < 0.001). This relationship persisted after adjustment (OR = 5.408, 95% CI: 2.827–10.344, *p* < 0.001).

Both good sleep quality and meeting WHO physical activity standards were independently associated with lower anxiety risk, and these relationships remained robust after controlling demographic and lifestyle factors.

### 3.4 Stratified analysis

To explore whether the effects of physical activity levels and sleep quality on anxiety differ across various demographic characteristics and health conditions, stratified analyses were conducted.

**Figure 4:**
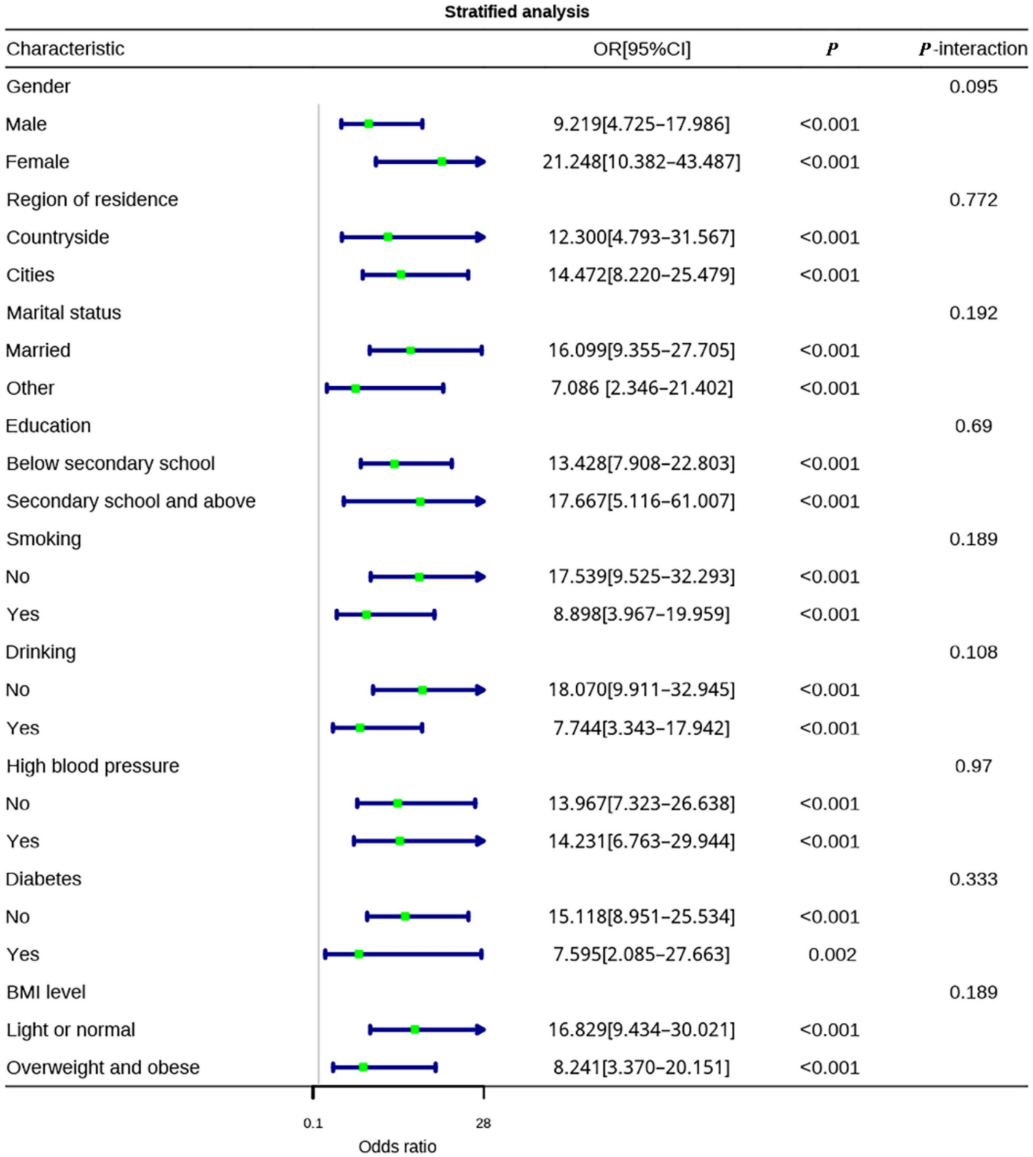
Stratified analysis of the impact of sleep quality on anxiety.

In the stratified analysis of sleep quality, it was found that the effect of sleep quality on anxiety was significant in all subgroups of demographic characteristics and health conditions (*p* < 0.05), with no statistically significant interactions (all *P*-interaction > 0.05). In the female subgroup, the protective effect of good sleep quality on anxiety (OR = 21.248, 95% CI: 10.382–43.487, *p* < 0.001) appeared numerically stronger than in the male subgroup (OR = 9.219, 95% CI: 4.725–17.986, *p* < 0.001). However, the interaction was not statistically significant (*P*-interaction = 0.095), and therefore, this observation should be interpreted with caution as it was not confirmed statistically. For residential area, the ORs were 14.472 (95% CI: 8.220–25.479, *p* < 0.001) in urban residents and 12.300 (95% CI: 4.793–31.567, *p* < 0.001) in rural residents; While the point estimate was slightly higher in urban participants, no statistically significant interaction was detected (*P*-interaction = 0.772), and the observed difference should not be overinterpreted. In the marital status subgroup, married individuals (OR = 16.099, 95% CI: 9.355–27.705, *p* < 0.001) showed a higher point estimate compared with those of other marital statuses (OR = 7.086, 95% CI: 2.346–21.402, *p* < 0.001), but the difference was not statistically significant (*P*-interaction = 0.192). No significant differences were found in the education level subgroup (*P*-interaction = 0.690), with non-smokers (OR = 17.539, 95% CI: 9.525–32.293, *p* < 0.001) and non-drinkers (OR = 18.070, 95% CI: 9.911–32.945, *p* < 0.001) showing stronger protective effects of sleep quality on anxiety compared to smokers (OR = 8.898, 95% CI: 3.967–19.959, *p* < 0.001) and drinkers (OR = 7.744, 95% CI: 3.343–17.942, *p* < 0.001), although the interaction was not statistically significant (Figure 4).

**Figure 5:**
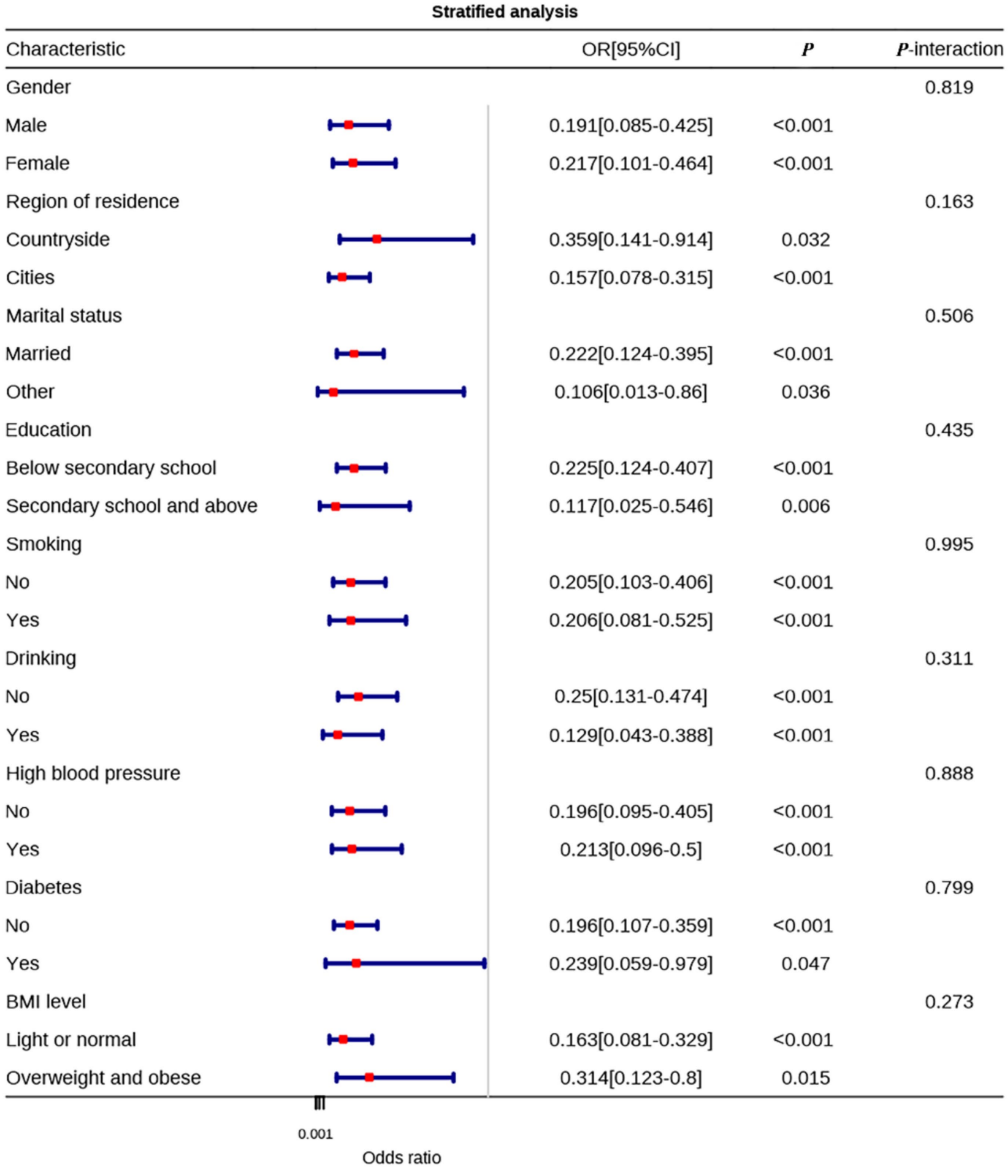
Stratified analysis of the impact of physical activity levels on anxiety.

In the stratified analysis of physical activity levels, the effect of physical activity on anxiety was significant in all subgroups (*p* < 0.05), with no statistically significant interactions (all *P*-interaction > 0.05). Unlike sleep quality, the effect of physical activity on anxiety did not differ significantly between males (OR = 0.191, 95% CI: 0.085–0.425) and females (OR = 0.217, 95% CI: 0.101–0.464) (*P*-interaction = 0.819). In the residential area subgroup, the protective effect of physical activity on anxiety was stronger in urban residents (OR = 0.157, 95% CI: 0.078–0.315, *p* < 0.001) compared to rural residents (OR = 0.359, 95% CI: 0.141–0.914, *p* = 0.032), but the interaction was not statistically significant (*P*-interaction = 0.163). In terms of marital status, the protective effect of physical activity on anxiety was stronger in married individuals (OR = 0.222, 95% CI: 0.124–0.395, *p* < 0.001) compared to those in other marital statuses (OR = 0.106, 95% CI: 0.013–0.86, *p* = 0.036), with no significant interaction (*P*-interaction = 0.506). No significant differences were observed in the education level, smoking status, drinking status, hypertension history, diabetes history, or BMI subgroups (all *P*-interaction > 0.05). Among non-drinkers, meeting physical activity standards had a stronger protective effect on anxiety (OR = 0.25, 95% CI: 0.131–0.474, *p* < 0.001) compared to drinkers (OR = 0.129, 95% CI: 0.043–0.388, *p* < 0.001). The protective effect of physical activity on anxiety was stronger in those with normal weight (OR = 0.163, 95% CI: 0.081–0.329, *p* < 0.001) compared to overweight or obese individuals (OR = 0.314, 95% CI: 0.123–0.8, *p* = 0.015), although these differences did not reach statistical significance (Figure 5).

The protective effects of good sleep quality and sufficient physical activity were consistent across demographic and health subgroups, and no statistically significant interactions were observed.

## 4 Discussion

This cross-sectional study examined the relationships between physical activity levels, sleep quality, and anxiety. The findings show that both poor sleep quality and insufficient physical activity are independently linked to an increased risk of anxiety, even after adjusting for potential confounders. Additionally, sleep quality, as measured by the PSQI, is a stronger predictor of anxiety than sleep duration.

Stratified analysis revealed varying patterns in the associations across demographic and health subgroups. While the interaction terms were not statistically significant, trends showed that good sleep quality had a stronger protective effect on anxiety in females and non-smokers. Similarly, meeting physical activity standards was more protective in urban residents, married individuals, and non-drinkers. These findings highlight potential subgroup differences that could guide tailored interventions. For instance, the stronger effect of sleep quality in females and non-smokers suggests that sleep improvement programs could benefit these groups. Additionally, the lower anxiety risk associated with meeting physical activity standards in urban residents suggests that environmental factors may play a role. These insights can inform more personalized public health strategies.

The relationship between sleep quality and anxiety in this study is consistent with previous research, while also providing new insights. Existing studies generally show a strong link between sleep disorders and anxiety (21). For instance, Zhou et al. found that both short sleep (<7 h) and long sleep (≥9 h) increased the risk of anxiety, supporting the idea that deviations from normal sleep duration can disrupt emotional regulation (5). However, this study refines this understanding by highlighting that sleep quality, as measured by the PSQI total score, is a more significant factor in anxiety than sleep duration alone. Poor sleep quality may affect neural circuits involved in emotional regulation, as research shows it reduces prefrontal cortex activity and increases amygdala activity, impacting emotional responses (22). Furthermore, disruptions in sleep stages may influence anxiety-related neural activity more than sleep duration (23). Individual differences, such as age or genetic factors, might explain why sleep duration alone did not significantly correlate with anxiety in this study.

This study confirmed the protective effect of meeting the WHO-recommended physical activity standards against anxiety, showing a strong link between adhering to these guidelines and reduced anxiety risk. However, the literature presents conflicting findings regarding the relationship between physical activity intensity and anxiety. Some studies suggest that high-intensity exercise improves mood, cardiovascular health, and energy levels, thus reducing anxiety (11, 24). In contrast, other research indicates that excessive high-intensity exercise can worsen anxiety, particularly due to elevated cortisol levels, fatigue, or overtraining (11). Individual differences, such as baseline anxiety, fitness level, and psychosocial stressors, may explain these conflicting findings. Additionally, the type of exercise (aerobic vs. resistance) plays a significant role, with aerobic exercises typically offering more consistent anxiety relief. This study did not differentiate between the effects of various physical activity intensities, an area warranting further exploration. Future studies should identify which intensities and exercise modalities are most effective for anxiety reduction, considering individual factors such as exercise preferences, baseline anxiety levels, and psychological views on exercise, which were not fully addressed in this study.

This study also found that the effects of sleep and physical activity on anxiety vary across demographic subgroups, suggesting that sociocultural factors may moderate this relationship. For instance, the protective effect of physical activity on anxiety was stronger in urban residents, likely due to the greater availability of structured exercise opportunities in urban areas. In contrast, the protective effect of sleep quality on anxiety was stronger in females, possibly linked to gender differences in neuroendocrine regulation (25). These findings expand on previous research and highlight the importance of considering demographic characteristics and social factors when designing strategies for anxiety prevention and intervention.

Physical activity can reduce anxiety through several biological mechanisms. It regulates neurotransmitter levels in the central nervous system, increasing the release of serotonin, dopamine, and endorphins, which are important for emotional regulation (26). Additionally, physical activity reduces systemic inflammation by lowering pro-inflammatory cytokines (IL-6, TNF-α, CRP), which are linked to the onset and progression of anxiety disorders (27). Regular exercise also boosts neurotrophic factors, such as brain-derived neurotrophic factors (BDNF), promotes hippocampal neurogenesis, and improves cognitive and emotional regulation (28). Poor sleep quality, on the other hand, may worsen anxiety through different pathways. Sleep disorders can overstimulate the hypothalamic–pituitary–adrenal (HPA) axis, leading to prolonged cortisol elevation, which is associated with heightened anxiety symptoms (11).

In addition to biological mechanisms, behavioral and psychosocial factors may also influence the relationship between sleep, physical activity, and anxiety. Good sleep and regular exercise can improve self-efficacy and coping skills, helping individuals better manage daily stress. Participation in physical activity, particularly group exercise, can also strengthen social connections and support, which are protective factors against anxiety. The urban–rural differences observed in this study may reflect the impact of environmental resources, such as access to exercise facilities and safe spaces. Urban areas typically offer more structured exercise opportunities (e.g., gyms, parks, community programs), while rural residents may engage more in labor-based physical activities, which can differ in both nature and psychological effects.

The findings have important implications for community-based mental health programs targeting middle-aged and older adults. Interventions such as sleep hygiene education, structured exercise, and access to safe exercise spaces should be incorporated into primary care and public health initiatives. The consistent associations across demographic subgroups suggest these interventions have broad applicability, while stratified patterns provide guidance for more tailored approaches for specific groups (e.g., females, non-smokers, urban residents). Additionally, including sleep and physical activity indicators in routine health screenings can help identify individuals at risk of anxiety, allowing for timely preventive interventions.

This study has several key limitations that should be considered when interpreting the results. First, the cross-sectional design prevents the establishment of causal relationships and cannot rule out reverse causality—anxiety may cause sleep disturbances or reduce physical activity, rather than the reverse. Future research should use prospective cohort designs or randomized controlled trials to clarify the direction of these relationships. Additionally, this study relied on self-reported questionnaires to assess sleep quality, physical activity, and anxiety levels, which may introduce bias, including social desirability bias. Future studies should incorporate objective measurement methods, such as accelerometer-based physical activity monitoring and polysomnography for sleep assessment, to improve the reliability of the findings.

## 5 Conclusion

This study provides valuable insights into the independent protective roles of good sleep quality and sufficient physical activity against anxiety in middle-aged and older adults. The findings suggest that both factors are strongly associated with a reduced risk of anxiety, independent of demographic variables and lifestyle factors. However, as a cross-sectional study, it is important to note that causality cannot be established, and these results should be interpreted as associations rather than causal relationships.

Despite the valuable insights, potential biases from self-reported measures must be acknowledged. The reliance on self-reported questionnaires for assessing anxiety, sleep quality, and physical activity may introduce reporting bias, as individuals may overestimate or underestimate their behaviors and symptoms. Additionally, the generalizability of these findings may be limited, as the sample was drawn from a specific geographic region and may not fully represent other populations, particularly younger adults or those from different cultural or socioeconomic backgrounds.

Future research should focus on longitudinal studies to better understand the temporal relationships between sleep quality, physical activity, and anxiety. Moreover, intervention studies are needed to test the practical applications of these findings, particularly to explore how improving sleep quality and increasing physical activity can reduce anxiety symptoms in real-world settings. These intervention trials will provide essential evidence to guide public health recommendations and mental health interventions.

## Data Availability

The original contributions presented in the study are included in the article/supplementary material, further inquiries can be directed to the corresponding author.
